# Comparison of the Efficacy and Safety of Sublingual Versus Oral Misoprostol for the Induction of Labor: A Randomized Open-Label Study

**DOI:** 10.7759/cureus.49422

**Published:** 2023-11-26

**Authors:** Mamta R Datta, Mousumi D Ghosh, Zainab AyazAhmed Kharodiya

**Affiliations:** 1 Obstetrics and Gynecology, Tata Main Hospital, Jamshedpur, IND; 2 Obstetrics and Gynecology, Tata Main Hospital, Manipal Tata Medical College, Jamshedpur, IND

**Keywords:** induction of labour, bishop score, induction to delivery time interval, sublingual misoprostol, oral misoprostol

## Abstract

Introduction

Misoprostol (prostaglandin E1 analog) is being used for the induction of labor by vaginal, oral, and sublingual routes. Oral misoprostol is the preferred route for induction of labor, but the use of sublingual misoprostol appears promising due to a faster onset of action. This study was done to compare the efficacy and safety of oral and sublingual misoprostol for induction of labor in term pregnancy.

Materials and methods

One hundred and sixty patients were randomly allocated to one of the two groups to receive 50 micrograms of oral and sublingual misoprostol four hourly for a maximum of six doses. Primigravida at 37-42 weeks of gestation with singleton pregnancy, cephalic presentation, Bishop score (<5), and reassuring fetal heart rate were included in the study. Misoprostol dose was withheld if the active phase of labor was reached or if the cervix was favorable for amniotomy (Bishop score greater than or equal to eight). The change in the Bishop score with misoprostol was studied along with adverse effects and neonatal outcomes.

Results

The mean number of 50 mcg misoprostol doses required was significantly less in the sublingual group (2.94±0.97 versus 2.13±0.92; p<0.0001). The rate of change of the mean Bishop score was faster in the sublingual group. After four hours of the first dose, the mean Bishop score changed to 3.52±2.14 versus 4.68±2.34 (p=0.001), and, similarly, after eight hours, it was 10.48±2.59 versus 11.39±2.06, and this difference was statistically significant (p=0.015). The mean induction delivery interval was significantly lower in the sublingual group. The need for labor augmentation, mode of delivery, and adverse effects were similar in both groups. The incidence of meconium-stained liquor and NICU admission was also similar in both groups.

Conclusion

Sublingmisoprostolstol has a short induction delivery interval and comparable side effects when compared to omisoprostolstol. Sublingmisoprostolstol is recommended for induction of labor at term.

## Introduction

Misoprostol is an oral prostaglandin compound structurally related to prostaglandin E1. It has the advantage of having a shorter induction to delivery time, higher vaginal delivery rate, lesser need for oxytocin, and a lower operative delivery rate when compared with other methods of induction, including oxytocin and vaginal and intracervical prostaglandin E2 [1, 2]. Misoprostol has advantages in being cheap, widely available even in most resource-poor settings, and remaining stable at room temperature. It is included in the World Health Organization (WHO) essential medicine list for several indications, including labor induction. Misoprostol is being increasingly used for the induction of labor by vaginal, oral, and sublingual routes. Orally administrated misoprostol has been shown to be an effective method for induction of labor in several publications. Vaginal misoprostol has been shown to be more efficacious than oral misoprostol in equivalent doses in some studies [3]. However, there has been the worry of excessive uterine contractility with vaginal doses of 50 µg or higher [4-6].

The dosage and route of administration of misoprostol have been evaluated in many studies in attempts to determine the appropriate dosing and best route of administration [7].

Oral misoprostol is commonly used for induction of labor, especially for pre-labor rupture of membranes. The use of sublingual misoprostol appears promising due to rapid absorption through sublingual mucosa and avoidance of first-pass metabolism [8,9], and, therefore, faster onset of action. This study was undertaken to compare the safety and efficacy of sublingual and oral misoprostol for induction of labor at term.

## Materials and methods

Study overview

The study was conducted in the Department of Obstetrics and Gynecology at Tata Main Hospital, Jamshedpur, Eastern India. Pregnant women admitted to Tata Main Hospital for induction of labor at 37-42 weeks of gestation comprised the study population. Only those women who fulfilled the inclusion criteria and were willing to participate in the study voluntarily were included in the study. An informed consent was taken prior to enrolment in the study. The study was done for a period of 10 months, from 1 December 2020 to 30 September 2021. This was a hospital-based, non-blinded comparative clinical study.

Study population

Inclusion criteria included primigravida admitted for induction of labor at 37-42 weeks of gestation with singleton pregnancies. Cephalic presentation, a Bishop score (<5), and a reassuring fetal heart rate were included in the study. Women with any contraindication to vaginal delivery, hypersensitivity to misoprostol, fetal anomalies, fetal growth restriction, genital bleeding, and chorioamnionitis were excluded from the study.

Sample size

Considering the overall prevalence of induction of labor as 20%, the total sample size needed to obtain an alpha error of 0.05 and the power of study of 87% was 160. The formula used for sample size calculation was: n = (z)2 p (1 - p) / d2, where n = sample size, z = level of confidence according to the standard normal distribution, p = estimated proportion of the population that presents the characteristic, and d = margin of error. Patients were randomly allocated to one of the two groups on the basis of random numbers generated by a web-based program.

Study procedure

**Figure 1:**
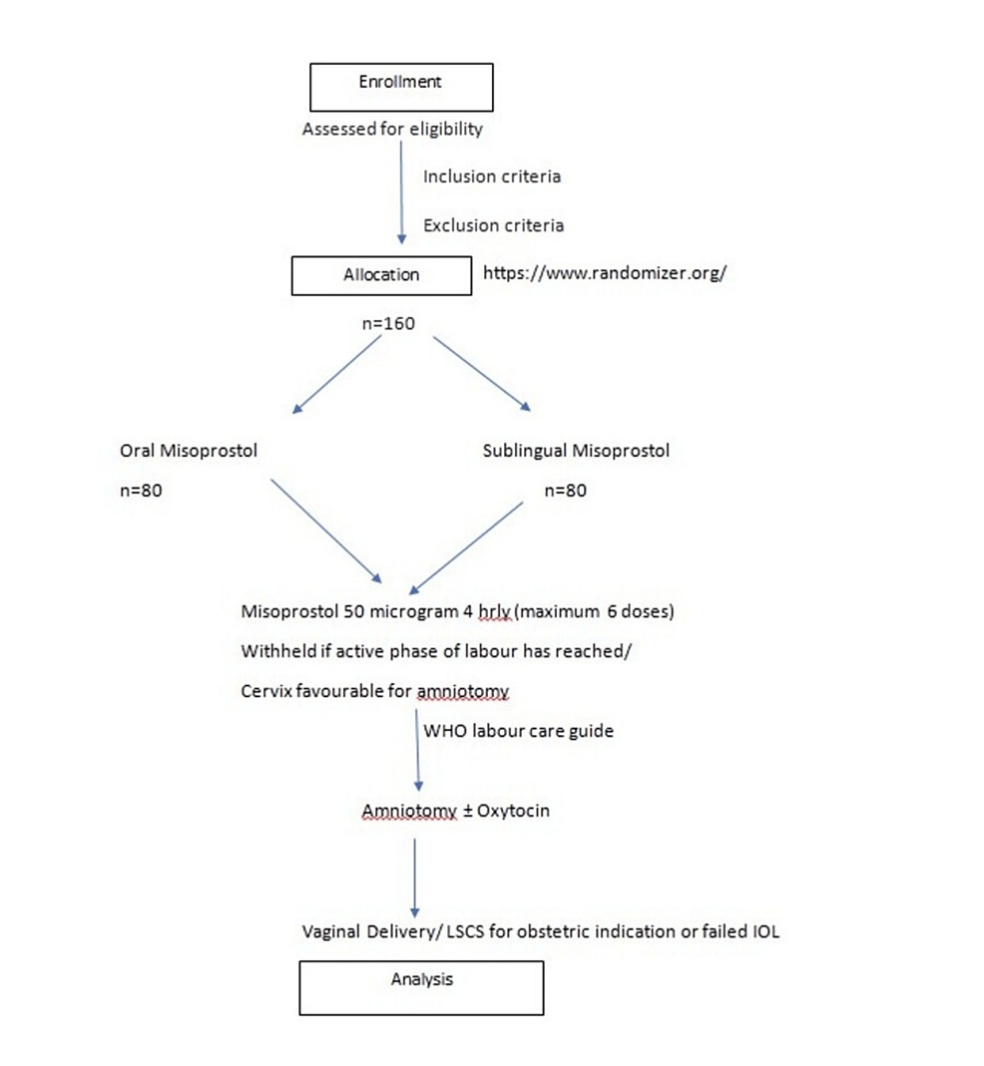
Consort diagram

On admission, a thorough history was taken, and a detailed general examination was done. After examination of the fundal height, lie, and presentation of the fetus, an ultrasound was done to evaluate the amniotic fluid index and confirm the findings. A pelvic examination was done to determine the bishop score and to rule out contracted pelvis. A nonstress test (NST) was done for 20 minutes, and if the NST was reassuring, the process of induction of labor was initiated. Women were randomly allocated to receive 50 microgram oral /sublingual misoprostol four hourly for a maximum of six doses. Misoprostol dose was withheld if the active phase of labor was reached (regular uterine contraction and cervical dilatation greater than 4 cm) or if the cervix was favorable for amniotomy (Bishop score greater than or equal to 8). As soon as fetal head engagement and cervical dilation permitted, an amniotomy was performed. Oxytocin augmentation was done if contractions were inadequate or the contraction pattern was dysfunctional. Oxytocin was not administered earlier than six hours after the last misoprostol dose, starting at 1 milliunit (mU)/minute and increasing by 1 mU/minute every 15 minutes until adequate contractions persisted. Two hourly fetal cardiotocography and intermittent fetal heart rate auscultation were done. Monitoring was done to look for tachysystole, hyperstimulation, and hypertonus. In such situations, the woman was advised to spit out the medication and wash her mouth. Labor induction was considered a failure if a woman did not enter the active phase of labor following six doses of misoprostol. The woman was then offered a cesarean section. The consort diagram is shown in Figure 1.

The primary outcome measure was the change of mean Bishop score with misoprost dosage. Secondary outcomes included the induction-to-delivery interval, the number of misoprostol doses administered, the need for augmentation of labor with oxytocin, maternal adverse effects, and neonatal outcomes.

Statistical analysis

For statistical analysis, data was entered into a Microsoft Excel spreadsheet (Microsoft, Redmond, Washington) and then analyzed by SPSS version 27.0 (SPSS Inc., Armonk, New York) and Graph Pad Prism version 5. 

## Results

The study was undertaken to compare the safety and efficacy of oral misoprostol (group A) and sublingual misoprostol (group B) for induction of labor in term pregnancy. A total of 160 women were enrolled in the study.

The two groups were comparable with respect to maternal age In group A, the mean age of women was 27.1± 3.8 years and in group B, the mean age of women was 27.2±4.4 years (p=0.9385). The mean gestational age was also comparable in both groups (38.48±1.06 weeks in group A and 38.68±1.05 weeks in group B; p=0.23). The indications for induction were gestational diabetes mellitus, obstetric cholestasis, premature rupture of membranes, postdated pregnancy and oligohydramnios. The two groups were comparable considering the indications for induction of labor. The preinduction Bishop score was similar in both groups. Forty-seven women in group A and 40 women in group B had a Bishop score of <3 (p=0.26), while 33 women in group A and 40 women in group B had bishop score between 3-6 (p=0.26; Table 1).

**Table 1:** Demographic characteristics Values are given as number (percentage) unless indicated otherwise

Variable	Group A (oral) n=80	Group B (sublingual) n=80	p-value
Age (years)			
18-20	3 (3.80)	6 (7.50)	0.16
21-25	25 (31.30)	20 (25.00)	0.29
26-30	37 (46.30)	34 (42.50)	0.62
31-35	15 (18.80)	20 (25.00)	0.23
Mean age in years (mean ± SD)	27.1 ± 3.8	27.2 ± 4.4	0.94
Mean gestation age in weeks (mean ± SD)	38.4 ± 1.06	38.6 ± 1.05	0.23
Indications for induction			
Gestational giabetes mellitus	15 (18.60)	17 (21.30)	0.62
Hypertensive disorder of pregnancy	18 (22.50)	18 (22.50)	1
Obstetric cholestasis	17 (21.30)	10 (12.50)	0.056
Premature rupture of membranes	16 (20.00)	19 (23.60)	0.47
Postdated pregnancy	5 (6.25)	6 (7.50)	0.67
Oligohydramnios with less fetal movement	9 (11.25)	10 (12.50)	0.7489
Preinduction Bishop score			
0-3	47 (58.75)	40 (50.00)	0.26
>3 -<6	33 (41.25)	40 (50.00)	0.26

We studied the change in the Bishop score in relation to the number of 50 microgram misoprostol tablets administered. When the first dose was given, the mean Bishop score was 3.32±1.11 in group A and 3.58±1.16 in group B (p=0.15). At the initial assessment, after four hours of the first dose, the mean Bishop score changed to 3.52±2.14 in group A and 4.68±2.34 in group B. This change was statistically significant. The mean Bishop score at eight hours (post two doses) was 10.48± 2.59 in group A and 11.39±2.06 in group B, and this difference was also statistically significant. Thus, sublingual misoprostol led to a faster change in Bishop score than oral misoprostol (Table 2).

**Table 2:** Misoprostol dosage and mean Bishop score change correlation

Time (hours)	Bishop score			
		Mean±SD	Median	p-value
0	Group A	3.32±1.11	3.00	0.1495
	Group B	3.58±1.16	3.50	
4	Group A	3.52±2.14	5	0.0013
	Group B	4.68±2.34	7	
8	Group A	10.48±2.59	11.00	0.0150
	Group B	11.39±2.06	12.00	

Five women (6.3%) in group A and 21 (26.3%) women in group B required only one dose (p=<0.0001) of misoprostol. Thus, significantly more women had successful induction with one dose of sublingual misoprostol. Thirty-three women (41.3%) in group A and 35 women (43.8%) in group B (p=0.7278) required two doses. Twenty-six women (32.5%) in group A and 18 women (22.5%) in group B required three doses (p=0.0872). Fourteen (17.5%) women in group A required four doses compared to only five women in group B (p=0.0035). Five doses were required in only two women in group A and one woman in group B. More than three doses were required in 16 women in group A compared to only six women in group B (p 0.011). The mean number of 50 mcg misoprostol doses required were significantly less in group B (2.94±0.97 in group A and 2.13±0.92 in group B; p<0.001). The median dose requirement was the same in both groups (Table 3).

**Table 3:** Dosage requirement of misoprostol Values are given as number (percentage) unless indicated otherwise

Dose requirement (50 mcg misoprostol)	Group A(Oral) n =80	Group B(Sublingual) n =80	Total	p-value
1	5 (6.30)	21 (26.30)	26	0.0006
2	33 (41.30)	35 (43.80)	68	0.7278
3	26 (32.50)	18 (22.50)	44	0.0872
4	14 (17.50)	5 (6.30)	19	0.0035
5	2 (2.50)	1 (1.30)	3	0.4122
Mean dose requirement (number of 50 mcg doses mean ± SD)	2.94 ±0.97	2.13 ±0.92	p<0.0001	
Median dose (no. of tablets)	3	3		

**Figure 2:**
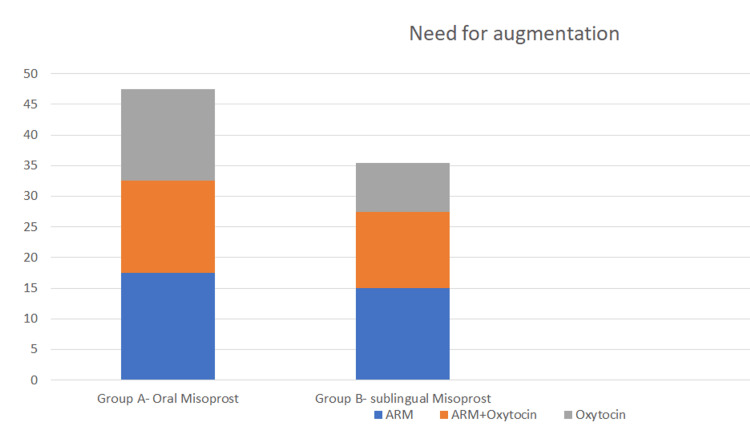
Need for augmentation ARM - artificial rupture of membrane

There was no significant intergroup difference in the number of women requiring artificial rupture of membranes (ARM), ARM with oxytocin and oxytocin alone. Fourteen women (17.5%) in group A and 12 (15.0%) in group B required augmentation with artificial rupture of membrane (p=0.58). Twelve women (15.0%) in group A and 10 (12.5%) in group B women were augmented with ARM and oxytocin(p=0.5485). Only oxytocin was used in 12 women (15.0%) in group A compared to seven women (8.8%) in group B (p=0.11). A total of 38 women in group A needed labor augmentation as compared to 29 women in group B (p=0.15). This difference was, however, not statistically significant. Oxytocin was used in 24 women (30%) in group A and in 17 women (21.25%) in group B (p=0.21). Thus, although a smaller number of women in the sublingual group required oxytocin for delivery, the difference was not statistically significant (Figure 2).

**Figure 3:**
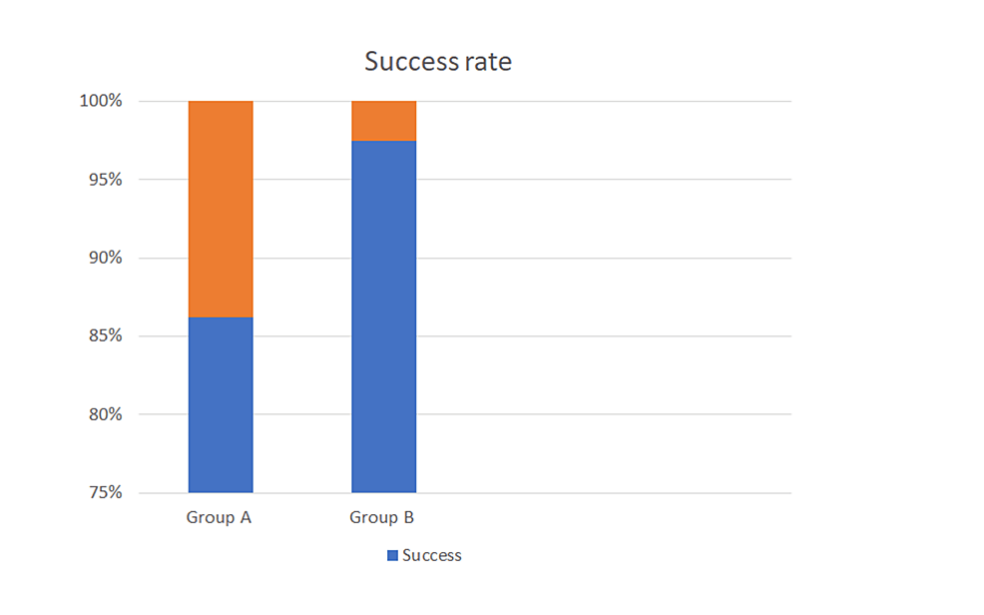
Success rate of misoprostol induction

The success rate was significantly higher in the sublingual group (86.2% in group A and 97.5% in group B; p=0.0045; Figure 3)

There was no difference in the mode of delivery in the two groups. Vaginal delivery rates were 56 (70.0%) and 64 (80%) in group A and B respectively (p=0.15; Table 4)

**Table 4:** Mode of delivery LSCS - lower segment cesarean section

Mode of delivery	Group A(Oral) n =80	Group B(Sublingual) n =80	p-value
	n (%)	n (%)	
Vaginal delivery	56 (70%)	64 (80%)	0.072
LSCS	24 (30%)	16 (20%)	0.072

The mean induction delivery interval in hours was significantly lower in group B (11.4± 4.7 versus 8.7± 4.4 hours, p=<0.001). 

Gastrointestinal effects like nausea, vomiting and diarrhea were similar in both groups (10 (12.5%) versus 6 (7.5%), p=0.15). The incidence of pyrexia was also similar in both groups (4 (5.0%) versus 2 (2.5%), p=0.1585). Only one (1.3%) woman had a headache in group A. The incidence of tachycardia was 1.3% in group A and 2.5% of women in group B (p=0.41). The incidence of tachysystole and hyperstimulation was similar in both groups. The overall incidence of side effects was higher in group A (p=0.19). This difference was not statistically significant. The mean birth weight was 2.87± 0.41 kg in group A and 2.91±0.49 kg in group B (p=0.5622). The birth weight was comparable in both groups. The incidence of meconium-stained liquor was 11.3% (9 of 80) in both groups. The NICU admission rate was also similar (10% (8 of 80) in group A and 6.3% (5 of 80) in group B; p=0.24). Only one baby had an Apgar score less than seven and this was in group B. No perinatal death was reported in the study group during the study period (Tables 5, 6).

**Table 5:** Maternal adverse effects GIT - gastrointestinal tract

Adverse effects	Group A (oral) n =80	Group B (sublingual) n =80	p-value
	n (%)	n (%)	
GIT effects	10 (12.5%)	6 (7.5%)	0.15
Pyrexia	4 (5.0%)	2 (2.5%)	0.25
Headache	1 (1.3%)	0 (0%)	0.15
Tachycardia	1 (1.3%)	2 (2.5%)	0.41
Tachysystole/ hyperstimulation	2 (2.5%)	1 (1.3%)	0.58
Total	16 (20.1%)	10 (12.5%)	0.19

**Table 6:** Neonatal outcome Values are given as number (percentage) unless indicated otherwise

Outcome	Group A (oral) n=80	Group B (sublingual) n=80	p-value
Meconium passage	9 (11.30)	9 (11.30)	1.00
NICU admission	8 (10.00)	5 (6.30)	0.24
Apgar score <7 at 5 minutes	0 (0.00)	1 (1.30)	0.16
Perinatal death	0 (0.00)	0 (0.00)	0.00
Total	17 (21.30)	15 (18.90)	0.69
Mean birth weight in kg (mean ± SD)	2.87 ± 0.41	2.91 ± 0.49	0.5622

Eleven women (13.8%) of group A and four women (2.5%) in Group B had meconium-stained liquor with fetal distress as the indication for cesarean delivery. Thus, the incidence of cesarean for fetal distress was higher in the oral group. The incidence of non-reassuring cardiotocograph (NRCTG) as an indication for cesarean delivery was similar in both groups (0.43; Table 7)

**Table 7:** Indications for cesarean delivery MSL - meconium-stained liquor; NRCTG - non-reassuring cardiotocograph

Fetal complications	Group A (oral) n=80	Group B (sublingual) n=80	p-value
	n (%)	n (%)	
Fetal distress + MSL	11 (13.80%)	4 (2.50%)	0.0044
NRCTG	13 (16.30%)	12 (15.33%)	0.4311

The incidence of postpartum hemorrhage in group A was 2.5% (2 of 80) compared to 3.8%(3 of 80) in group B (p=0.6495). One woman had a cervical tear in group B. Overall, third-stage complications were seen in two women in group A and four women in group B (p=0.28). This difference was not statistically significant (Table 8)

**Table 8:** Third stage of labor complications Values are given as number (percentage)

Complications of third stage of labor	Group A (oral) n=80	Group B (sublingual) n=80	p-value
Postpartum hemorrhage	2 (2.5%)	3 (3.8%)	0.6495
Cervical tear	0 (0%)	1 (1.3 %)	
Complete perineal tear	0 (0%)	0 (0%)	
Vulval hematoma	0 (0%)	0 (0%)	
Total	2 (2.5%)	4 (4.11%)	02844

## Discussion

This study compared the safety and efficacy of the oral and sublingual routes of administration of misoprostol for induction of labor in term pregnancy. The participants in the two arms of the study were comparable with respect to age, parity, preinduction Bishop scores, and the indications for induction of labor. We found very few studies that directly compared oral and sublingual misoprostol as methods for induction of labor in term pregnancies.

Caliskan et al. [10] reported that the mean number of 50 mcg misoprostol doses required for induction in his study was 1.9±1.2 in the sublingual group. Parimkayala et al. [11] also reported that the sublingual group had a lesser number of women requiring more than one dose of 50 mcg misoprostol compared to the oral group. Three out of 19 (15.7%) women in the sublingual group delivered vaginally by a single dose of misoprostol in this study. Ifariola et al. [12] also reported that the mean number of doses of sublingual misoprostol for successful induction was 1.26±0.44 in their study. Malik et al. [13] studied 100 primigravida with singleton pregnancy at term with pre-labor rupture of membranes and unfavorable Bishop score. They used 100mcg misoprostol administered either orally or sublingually at four hourly intervals for a maximum of two doses. Regarding dosage, 64% of women delivered with a single dose in group B, while only 32% delivered with a single dose in group A (p<0.05). We have also reported a lower mean number of misoprostol doses in the sublingual group as compared to the oral group. The mean number of 50mcg doses was 2.49±0.97 in the oral group and 2.13±0.92 in the sublingual group in our study (p=<0.0001). Twenty-one of 80 (26.3%) women delivered with a single dose of sublingual misoprostol as compared to five of 80 (6.3%) women in the oral group (p=0.0006) in our study.

Parimkayala et al. [11] reported that 46.7% of women required oxytocin in the sublingual group compared to 75% in the oral group. This difference was statistically significant. Deepika et al. [14] compared sublingual misoprostol with intracervical dinoprostone gel and reported that there was a significant reduction in the requirement of augmentation by artificial rupture of membranes (ARM) in the group receiving sublingual misoprostol. Only 17% of women required oxytocin in the sublingual group in their study. The dose used in this study was 100 mcg. Shetty et al. [15] reported that 58.2% of women required oxytocin augmentation with oral misoprostol. Parimkayala et al. [11] reported that 46.7% of women required oxytocin in the sublingual group compared to 75% in the oral group. Kalra et al. [16] also reported that only 35% of women in the sublingual group required oxytocin augmentation. We have reported that the need for augmentation and oxytocin requirements were similar with oral and sublingual routes (30% vs. 21.25%; p=0.2064).

Shetty et al. [15] reported a 0.8% incidence of uterine hyperstimulation in the oral group. Caliskan et al. [10] reported that the proportion of women who experienced uterine tachysystole or chorioamnionitis did not significantly differ by route of administration. Bartusevicius et al. [17] compared vaginal and sublingual misoprostol and reported that the incidence of tachysystole was more than three-fold higher in the sublingual than in the vaginal group, but this was not statistically significant. Ayati et al. [18] reported that the maternal complication in the sublingual group included residual placenta (2%), tachysystole (2%), vomiting (12%), atony (3.3%) and abdominal pain (5.5%), although there was no significant difference between two groups (vaginal and sublingual). Dorr et al. [19] also reported that the proportion of women who experienced uterine tachysystole or chorioamnionitis did not significantly differ by route of administration. Perveen et al. [20] compared oral, sublingual, and vaginal misoprostol for cervical ripening for first-trimester abortion. The sublingual group had significant cervical dilatation, and the side effects were less as compared to the vaginal and oral routes. Loose motions and nausea/vomiting were more with the sublingual and oral routes, while blood loss was more in the vaginal route. Wallstrom et al. [21] reported that oral and sublingual misoprostol had similar incidences of postpartum hemorrhage. We have reported comparable side effects and third-stage complications with both routes of misoprostol administration. We have reported that the incidence of maternal adverse effects, neonatal outcome, and third stage labor complications were comparable with both routes of administration of misoprostol.

The vaginal delivery rate with both routes of misoprostol administration was comparable in our study (70% vs. 80%, p=0.15). However, we reported a higher incidence of fetal distress as an indication of cesarean delivery with the oral route. Shetty et al. reported that 46.3% of women delivered within 24 hours. Deepika et al. [14] reported a vaginal delivery rate was 66% with sublingual misoprostol. Dorr et al. [19] did a retrospective analysis of 207 women receiving either vaginal or buccal misoprostol. They reported a vaginal delivery rate of 84.9% and failed induction rate of 5.37% in the sublingual group (5 of 93). Ayati et al. [18] compared the efficacy and safety of vaginal versus sublingual misoprostol for cervical ripening and induction of labor in 140 women using 25mcg of misoprostol. They reported 85.9% vaginal delivery rate (vaginal + vacuum delivery) and 71.8% delivered with only two doses of sublingual misoprostol in their study.

Bartusevicius et al. [17] compared the efficacy and safety of 50μg of sublingual misoprostol with 25μg of vaginal misoprostol administered for labor induction at term in a double-blinded, randomized controlled trial on 140 women at term for labor induction. Fifty-eight women (83%) in the sublingual misoprostol group delivered vaginally within 24 hours. Siwach et al. [16] studied 160 women randomized to receive 25mcg of oral misoprostol given three hourly and 25mcg of sublingual misoprostol given four hourly. They reported a comparable number of women delivering within 24 hours of induction in both groups (93.1 % vs. 83.7%). Bansal et al. [22] reported an 86% vaginal delivery rate with sublingual misoprostol, with 50% delivering within 12 hours of induction of labor. Malik et al. [13] reported that in the sublingual misoprostol group, 92%of women delivered within 12 hours of induction, while 80% of subjects delivered in this time period in the oral group, p<0.05). There was no failed induction in either group. However, they used 100mcg doses in their study for a maximum of 2 doses given at four hours intervals. The rate of cesarean section was 8% in the sublingual group and 20% in the oral group, which is statistically insignificant. 

Shetty et al. [15] reported that the mean induction to vaginal delivery interval was 27.9 hours in the oral group. Ayati et al. [18] reported an induction to delivery interval of 11.62±6.76 hours with 25mcg sublingual misoprostol. Deepika et al. [14] reported that the misoprostol group showed shorter induction to delivery interval, a greater number of vaginal deliveries, and a reduced need of cesarean sections compared to the dinoprostone group. In their study, the induction to delivery interval was 14.1 hours with oral misoprostol. Parimkayala et al. [11] showed that oxytocin induction to vaginal delivery time was < 24 hours in 43 (71.7%) in the sublingual group and 36 (60%) women delivered vaginally in <24 hours in the oral group. No significant difference was found in the number of women delivering vaginally within 24 hours of induction among both the groups in their study. Ayati et al. [18] reported an induction to a delivery interval of 11.62±6.76 hours with sublingual misoprostol. Bartusevicius et al. [16] reported an induction to delivery interval of 15.0±3.7 hours in the sublingual group. Ifariola et al. [12] reported that the induction‑delivery interval was 10.02±2.37 hours in the sublingual group. Mahdi et al. [23] conducted a retrospective study of 2404 women with oral and sublingual misoprostol over a period of five years. They concluded that sublingual misoprostol was associated with a significantly shorter induction to vaginal delivery time. However csarean delivery rate was lower in primiparous women induced with oral misoprostol compared to sublingual. This report is contrary to the prospective study results reported in the literature and our study results. This may be due to the difference in the oral dosage of misoprostol.

Shetty et al. [15] found no significant differences in neonatal outcomes in two groups, i.e., oral and sublingual. Malik et al. [13] also found no significant difference in fetal outcomes in their study of oral and sublingual misoprostol. However, Deepika et al. [14] reported a NICU admission rate of 20%. In our study, the neonatal outcomes were comparable in both groups, with NICU admission of only 10% in the oral group and 6.3% in the sublingual group (p=0.24).

The strength of our study is the prospective design, where the patients in both arms were followed from induction to delivery. The limitation of the study was that it was an open-labeled trial, and blinding could not be done. Another constraint was the short duration of the study with a limited sample size.

## Conclusions

Sublingual misoprostol is a safe, effective, and acceptable method compared to oral misoprostol for induction of labor in term singleton pregnancy with unfavorable cervix. Sublingual misoprostol has a short induction delivery interval with comparable vaginal delivery rates, neonatal adverse outcomes, and maternal side effects. Sublingual misoprostol is recommended for induction of labor at term. However, larger multicentric studies need to be undertaken to validate our results.
